# Development and Validation of an Explainable Prediction Model to Assess the Risk of Coronary Artery Disease in Young and Middle-Aged Individuals

**DOI:** 10.31083/RCM39006

**Published:** 2025-09-23

**Authors:** Haolin Shi, Shanshan Zhao, Yingshuai Wang, Chongyang Zhang, Yanli Wan

**Affiliations:** ^1^Institute of Medical Information/Library, Chinese Academy of Medical Sciences & Peking Union Medical College, 100020 Beijing, China

**Keywords:** coronary artery disease, young and middle-aged adults, risk assessment, machine learning

## Abstract

**Background:**

There is currently a lack of adequate risk assessment for coronary artery disease in the young and middle-aged population (ages 20–60). This cohort is characterized by limited symptom presentation, low utilization of medical facilities, and challenges in accessing healthcare services. Consequently, these individuals experience difficulties in early disease identification, rendering them susceptible to sudden cardiac death and premature mortality upon the manifestation of symptoms. Data from regular blood and urine tests, as well as questionnaires, are readily available and well-documented across diverse healthcare environments. Hypertension is a notable risk for coronary artery disease within this population. In light of these challenges, we present a risk assessment system for coronary heart disease specifically tailored for young and middle-aged individuals with hypertension, utilizing data derived from blood and urine examinations in conjunction with a brief questionnaire.

**Methods:**

The dataset was sourced from the National Health and Nutrition Examination Survey (NHANES) database, covering the years 2005–2019. Following three iterations of feature selection, we identified 26 pertinent features. Subsequently, we developed five predictive models to facilitate large-scale screening for coronary heart disease risk. To enhance the interpretability of our models, we employed SHapley Additive exPlanations (SHAP) to evaluate the individual contributions of each feature.

**Results:**

We included 709 patients diagnosed with coronary artery disease and 6409 healthy individuals in our analysis. The results showed that LightGBM exhibited the highest performance (area under the curve (AUC) of 0.93).

**Conclusions:**

This study has the potential to facilitate the improved screening of patients with coronary artery disease; we have developed a risk assessment system that is freely accessible to the public: https://prediction-of-coronary-heart-disease-htn-young-adults.streamlit.app/.

## 1. Introduction

Coronary artery disease (CAD) has traditionally been associated with older populations; however, recent trends indicate an increasing incidence among individuals under 60. While advancements in medical care have contributed to decreased mortality rates from cardiovascular disease (CVD) from 2003 to 2013, it still poses a substantial risk of illness and death for young and middle-aged people. Daily, over 2000 Americans die from CVD, with a significant proportion of these individuals being under the age of 65. Once CAD develops in middle-aged or young adults, they become vulnerable to myocardial infarction (MI) and may face premature mortality [1]. Young adults are at a notably high risk of developing CVD. While the occurrence of acute coronary syndromes (ACS) has decreased in older age groups, there has not been a corresponding decrease in cardiovascular events among young men and women, especially those experiencing acute MI [2]. In 2019, approximately 17.9 million individuals in the United States succumbed to acute myocardial infarction (AMI). In addition, there has been a rise in the occurrence of AMI among young and middle-aged individuals. Hospitalizations for AMI in this age group rose from 27% between 1995 and 1999 to 32% between 2010 and 2014. There is a lack of adequate literature on coronary heart disease and MI in those who are young or middle-aged, specifically, those who are 60 years of age or younger, yet the consequences of MI can be disastrous, significantly impacting mental health, work capability, and socioeconomic conditions [3]. Thus, early screening for CAD in young and middle-aged individuals is an important tool for secondary prevention.

Despite advancements in diagnosis and treatment table progress in diagnosing and treating CAD, challenges remain in effectively addressing the condition in the younger population. These challenges include atypical and delayed symptoms, lack of compliance with therapy, and distinct syndromes related to age [4]. Prodromal symptoms (PS) are temporary sensations that occur in the days, weeks, or months leading up to a heart attack. These symptoms can be either particular or vague [5]. PS are distinct or indistinct temporary sensations that occur several days or months before a heart attack. Regrettably, young, and middle-aged individuals frequently disregard these cautionary indicators. While 72% of men and 85% of women aged 55 or younger encounter PS before a heart attack, only 41.9% of men and 49.9% of women seek medical treatment during this period [6]. In general, the clinical manifestation of MI in young and middle-aged individuals is comparable to that of older patients. Many of these patients have chest discomfort because of plaque rupture. Nevertheless, a prior occurrence of angina before a myocardial infarction is infrequent, occurring in only 25% of these individuals [7]. Young individuals face restricted access to healthcare resources, making it difficult to detect, diagnose, and treat CAD before it develops. Consequently, doing early screening for coronary heart disease at basic healthcare institutions or at medical check-ups, which are more easily reachable, could aid in the prevention of myocardial infarction. These institutes regularly offer blood and urine testing, as well as collect basic questionnaire data. Hence, the creation of a CAD risk assessment model for young individuals with this data could be used to reduce the risk of myocardial infarction and enhance the adoption of screening.

Individuals who are young or middle-aged and who have high blood pressure may face a slightly increased likelihood of experiencing cardiovascular problems in the future. Notably, the population-attributable proportion of cardiovascular events linked to high blood pressure is higher in younger individuals compared to older adults with similar blood pressure values. This finding suggests that hypertension has a more detrimental effect on cardiovascular events in young and middle-aged individuals, especially when blood pressure levels are above 140/90 mm Hg [8]. This is especially true for individuals with multiple comorbidities or risk factors. For those with hypertension but fewer health conditions, elevated blood pressure plays a significant role. Thus, it would be beneficial to focus resources on high-risk groups, such as individuals with hypertension, to enhance cost-effectiveness in screening asymptomatic young and middle-aged individuals for CAD.

Coronary angiography and fractional flow reserve (FFR) are well-established gold standards for diagnosing CAD; however, these are invasive procedures. Many non-invasive tests are now available for CAD diagnosis, with coronary computed tomography angiography (CTA) and FFR computation from CTA datasets (FFRCT) recognized as established tools [9]. However, arrhythmias can affect the accuracy of CTA diagnostic results [10]. Given the constraints of economic resources and availability, there is a strong demand for a cost-effective and non-invasive screening tool to identify CAD in young individuals with hypertension, before a formal diagnosis is made. In recent years, many machine learning models have been developed to predict CAD risk using a variety of different data [11]. For instance, Forrest IS *et al*. [11] created a model based on electronic health records (EHR) that outperformed better than Pooled Cohort Equation (PCE) in predicting the status of CAD after one year.

Several CAD risk assessment models are currently available: the Framingham Risk Score (FRS), the PCE, the Polygenic Risk Score, the Systematic Coronary Artery Risk Evaluation (SCORE2), and the China-par 10-year risk prediction model. The FRS is the first risk prediction model used in North America to estimate cardiovascular risk and guide statin therapy for primary prevention. They incorporate six risk factors: age, total cholesterol, weight, electrocardiograph (ECG), smoking, and systolic blood pressure. Concerns regarding the FRS potentially overestimating risk and its lack of generalizability to the current US population led to the PCE being created utilizing data from five different cohorts across the United States [12]. The PCE model includes additional parameters such as high-sensitivity C-reactive protein, apolipoprotein B, glomerular filtration rate, microalbuminuria, family history, cardiorespiratory fitness, ankle-brachial index, carotid intima-media thickness, and coronary artery calcium score, in addition to the standard risk factors. To date, over 1790 CAD loci have been discovered through extensive genome-wide association studies (GWAS) [13]. Recent data suggest that the aggregation of these common variants into risk scores (called genetic risk scores or PRS) can improve CAD risk prediction and stratification [14]. SCORE2 calculates the 10-year risk of CVD in both men and women from four different regions in Europe and has been endorsed for use in clinical practice by the 2021 European Society of Cardiology (ESC) Guidelines for the Prevention of CVD [15]. The data utilized in SCORE2 includes traditional CVD risk factors such as age, smoking status, blood pressure, cholesterol, and high-density lipoprotein (HDL) levels, along with diabetes-specific factors, including age at diagnosis, blood glucose levels, and renal function. The China-prediction for atherosclerotic cardiovascular disease (ASCVD) risk in China (PAR) project successfully developed and verified the initial equation for predicting the 10-year ASCVD risk in the Chinese population by analyzing data from four extensive, up-to-date, population-based Chinese cohorts. These risk prediction equations serve as a significant tool for accurately measuring risk and directing personalized primary care in Chinese populations [16]. The system includes additional significant risk factors, incorporating four supplementary variables: Waist Circumference, geographic region, urbanization, and family history of ASCVD, along with primary risk factors such as age, treated or untreated systolic blood pressure (SBP), total cholesterol, high density lipoprotein cholesterol (HDL-C), current smoking, and diabetes. While these models have been commonly used, it is important to note that the risk factors for CAD in young and middle-aged populations differ from those in older persons. As a result, these models are not designed to apply to individuals between the ages of 20 and 60, and their application may lack generalizability to early screening in primary healthcare settings or medical examination facilities.

However, most existing risk models have been developed using data from older populations, which may limit their ability to reflect the unique risk profiles of younger individuals with hypertension. To address this gap, the present study seeks to develop and validate a machine learning-based risk assessment tool specifically for young and middle-aged adults with hypertension. Hence, this study utilized the National Health and Nutrition Examination Survey (NHANES) 2005–2019 dataset to examine the risk of CAD and the significance of early screening for hypertension in young and middle-aged individuals. Pre-processing was conducted on the data, and feature selection was carried out to identify 26 risk factors for CAD from the questionnaire and routine blood and urine examination data provided by NHANES. Afterward, five machine learning algorithms were utilized to create a risk assessment model for CAD in young and middle-aged persons with hypertension. Furthermore, to understand the model, significant risk variables for hypertension were determined using SHapley Additive exPlanations (SHAP).

## 2. Methods

### 2.1 Study Design and Participants

NHANES is a health-oriented program in the United States. The Centers for Disease Control (CDC) and the National Center for Health Statistics (NCHS) perform this health survey quarterly. The study evaluates the intricate health condition of Americans through a variety of sophisticated stratified, multistage sampling techniques. The data is made available to the public at no cost for research purposes. We utilized NHANES data gathered between 2005 and 2019, including a total of 43,411 participants. Exclusion criteria were (1) age >60 years and age <20 years. (2) Participants with missing or ambiguous information regarding the presence or absence of coronary artery disease. (3) Variables with more than 10% missing data across all participants were excluded from the analysis. The inclusion criteria consisted of individuals who had hypertension. Following the process of inclusion and exclusion, a total of 7118 individuals between the ages of 20 and 60 were selected for the analyses. These analyses encompassed several factors such as demographic information, blood pressure measures, body measurements, laboratory tests of interest, and pertinent questionnaire data. All these assessments were conducted at the mobile examination center (MEC). Refer to **Supplementary Fig. 1** for a flow chart illustrating the screening process of the study population.

Biological sample collection was performed at the MEC. Any variable with a missing value of more than 50% was not considered for analysis, and test results for a total of 51 chemicals in the blood and urine of interest were collected from the remaining NHANES data from 2005–2019, including basophil count, cadmium, lead, eosinophil count, fasting glucose (mmol/L), HDL, low-density lipoprotein (LDL), lymphocyte count, monocyte count, neutrophil count, albumin, blood urea nitrogen, total calcium, cholesterol, creatinine, globulin, glucose (serum), iron, phosphorus, total bilirubin, total protein, triglycerides, uric acid, total cholesterol, mercury, percentage of basophils (%), cadmium, percentage of eosinophils (%), fasting glucose (mg/dL), erythrocyte pressure (%), hemoglobin, percentage of lymphocytes (%), mean cellular hemoglobin concentration, mean cellular hemoglobin, mean cell volume, percentage of monocytes (%), mean platelet volume, percentage of neutrophils (%), platelet count (%), erythrocyte count, erythrocyte distribution width (%), alkaline phosphatase, aspartate transaminase (AST), alanine transaminase (ALT), bicarbonate, chloride, gamma glutamyl transferase, potassium, lactate dehydrogenase, osmolality, white blood cell count.

The selected questionnaire data were variables with known risk factors for coronary heart disease, including alcohol consumption, blood pressure, cholesterol, diabetes mellitus, income, medical conditions, kidney, depression, occupation, oral health, physical activity, sleep disorders, smoking, and weight history.

### 2.2 Assessment of Hypertension and Coronary Artery Disease

Any of the following factors qualify a participant as having high blood pressure:

(1) When asked if they had ever been told by a doctor or other healthcare provider that they had high blood pressure (often referred to as hypertension), they answered “yes”.

(2) When asked if they were currently taking medication for hypertension or if they were also taking anti-hypertensive medication, they answered “yes”.

(3) We used the average of three blood pressure readings to define hypertension (mean SBP 140 mm Hg and/or diastolic blood pressure (DBP) 90 mm Hg); if blood pressure measurements were interrupted or insufficient, the average was taken after the fourth reading.

The assessment of coronary heart disease status relied heavily on questionnaires, and a yes answer to the following questions was taken as indicative of the presence of coronary heart disease in the subject:

(1) Participants were asked if a healthcare professional had ever diagnosed them with coronary heart disease.

(2) Participants were asked if a healthcare professional had ever diagnosed them with angina.

(3) Participants were asked if a healthcare professional had ever diagnosed them with a heart attack.

### 2.3 Other Data Selection and Measurements

Demographic characteristics encompass various factors such as gender, age, pregnancy status (including pregnant, not pregnant, or undeterminable), country of birth (including the 50 U.S. states, Washington, D.C., Mexico, or other Spanish-speaking countries), education level (ranging from less than 9th grade to high school graduate, some college or AA graduate or higher), marital status (including unmarried, married or living with a partner, married but currently living alone, or divorced or widowed), and the country of birth of the family reference.

For the diagnosis of depression, we used the Patient Health Questionnaire (PHQ)-9 [17]. The PHQ-9 consists of 9 items based on the Diagnostic and Statistical Manual of Mental Disorders IV criteria for the diagnosis of depression. Each item rates the frequency of depressive symptoms on a 3-point scale (0 = “not at all” to 3 = “almost every day”). Scores range from 0 to 27, with higher scores indicating greater severity of depression.

### 2.4 Statistical Analysis

Descriptive statistics were used to distinguish between participants with and without hypertension. The baseline information was presented using averages and standard errors for continuous variables, and percentages and counts for categorical variables. Between-group disparities were evaluated using *t*-tests for continuous variables and chi-square tests for categorical variables. NHANES utilizes a stratified, multistage probability sampling method, in which each participant is given a specific sampling weight determined by the primary sampling unit. Nevertheless, this study utilized unprocessed and unadjusted data from NHANES to develop machine learning and deep learning models. Weighted data is not used because it is normally used to estimate nationwide incidence and prevalence rates. The estimation of national prevalence was unnecessary; the primary objective was to examine the correlation between coronary heart disease and individual variables to train the model.

Our research was conducted using Python 3.12 (Python Software Foundation, Beaverton, OR, USA) and compatible open-source packages for data analysis and machine learning (ML) model building.

### 2.5 Feature Preprocessing and Selection

To obtain high-quality data, the initial data were pre-processed. Outliers in the original data were defined as values below Q1 – 1.25 interquartile range (IQR) or above Q3 + 1.25 IQR, and these outliers were replaced with the median. This threshold was chosen to strike a balance between sensitivity to extreme values and preservation of meaningful variability in clinical data. Datasets with fewer than 50% missing data were divided into two groups: one with CAD and one without. Categorical variables were imputed using the mode. For continuous variables, missing values were imputed using random sampling from the observed (non-missing) values within the same group, a method that preserves the original distribution and avoids artificial smoothing. The categorical variables were transformed using one-hot encoding, while the continuous variables were scaled to a normalized range of 0 to 1.

Feature selection was used to reduce redundant features that could negatively impact model performance, as not all features contained meaningful information. Univariate logistic regression analyses were performed for each variable, and variables that were statistically significant with a *p*-value less than 0.05 were chosen for additional feature selection.

This study utilized a filtering strategy to identify features.

(1) Step 1: Akaike Information Criterion (AIC)-based Stepwise Backward Elimination. The initial stage entailed a systematic process of eliminating features using stepwise backward feature selection, which was guided by the AIC. The method iteratively removes features with less information. It balances model complexity and goodness-of-fit and helps to retain variables that contribute significantly to the model.

(2) Step 2: Correlation filtering. To reduce multicollinearity, we calculated Pearson’s correlation coefficient (PCC) among the features retained after Step 1. When two features showed moderate correlation (r > 0.4), the feature with the higher area under the curve (AUC) was kept. The threshold of 0.4 was chosen as a conservative value to minimize redundancy, while still preserving predictive strength.

(3) Step 3: Incremental feature selection (IFS). The third stage entails utilizing the IFS technique to choose the most suitable set of features. The IFS strategy prioritizes the addition of features based on their significance and generates many subsets of features. This step ensures that the most predictive combination of features is chosen without overfitting.

### 2.6 CAD Risk Assessment Model

This paper utilizes five classification models for comparison: multilayer perceptron (MLP), XGBoost, LightGBM, CatBoost, and random forest. These algorithms each have distinct characteristics. Random forests, first introduced by Breiman in 2001, consist of collections of classification and regression trees. These trees are constructed using randomly selected training datasets and random subsets of predictor variables. Random forests are simple models that use binary splits of predictor variables to determine outcome predictions [18]. Gradient boosting (GB) is a member of the ensemble learning paradigm that constructs trees through iterative refinement [19]. In each iteration, the current tree is built upon the previous tree, with the difference between predicted and actual values calculated and used as the target for the current tree. Subsequently, after hundreds of iterations, the differences are gradually minimized, and the results from all the trees are aggregated to produce the final prediction. XGBoost is a tree-based model characterized by its efficient and flexible handling of missing data and its ability to combine weak predictive models to create accurate predictions [20]. LightGBM, a variant of GB developed by Microsoft, has demonstrated excellent performance in processing very large, structured datasets [21]. CatBoost is also a tree-based model that delivers state-of-the-art performance on structured tabular data with rapid training times [22]. An MLP typically comprises three layers: an input layer, a hidden layer, and an output layer. Earlier, MLP determined connection weights between layers through error correction learning. With the introduction of the BP algorithm, MLP can now adjust connection weights iteratively, layer by layer [23]. The MLP network employed in this study comprises three fully connected layers: two hidden layers and one output layer. The activation function selected was “ReLU”, and the “RMSprop” optimizer was used to estimate gradients for gradient descent. This approach was employed to optimize the network and save the model with the highest validation accuracy during training.

Due to the extreme sample imbalance problem in this study, we applied Synthetic Minority Over-sampling Technique (SMOTE) to generate synthetic samples, as well as downsampling and other techniques. However, none of these methods led to an improvement, and some even resulted in a decrease in the predictive performance of the models. Consequently, we adjusted the positive sample weights, setting the positive-to-negative sample weight ratio to 2:1 in all models, except for MLP.

The dataset was partitioned into a training set, used for constructing the model, and an internal validation set, at an 8:2 ratio. To enhance the accuracy of evaluating the model’s performance, we utilized ten-fold cross-validation. Furthermore, we conducted hyperparameter optimization (HPO) using genetic algorithms (GA) for these five classification models to select the model with the highest accuracy. The GA was configured to search a predefined parameter space including num_leaves, max_depth, learning_rate, n_estimators, and min_child_samples. The optimal parameter combination was determined based on the highest average accuracy across the cross-validation folds. The model’s performance was assessed by analyzing the AUC of the individuals’ operating characteristics, as well as their accuracy, precision, specificity, and F1 scores.

### 2.7 Variable Importance

We use the Gini index to calculate feature importance and compare it with the feature importance rankings from random forests (RF) and LightGBM. For model interpretation, we use SHAP values to elucidate how different machine learning models operate. Positive SHAP values indicate that features contribute positively to the prediction, whereas negative values signify negative contributions [24]. The SHAP value for each feature is calculated for each sample to illustrate the importance of each feature more clearly in our prediction model.

The SHAP method offers both global and local interpretations for model analysis. Global interpretation provides consistent and accurate attribution values for each feature, highlighting associations between input features and CAD. Local interpretations offer insights into specific predictions for individual cases based on their data.

### 2.8 Webpage Deployment Tool Based on the Streamlit Framework

We developed a web application using Python’s Streamlit framework. By inputting the 26 features included in the final model, the application returns the probability of CAD and a force plot of individual feature contributions.

## 3. Results

### 3.1 The Baseline Characteristics of the Study Population

After preprocessing the data, we compiled a dataset comprising 709 individuals diagnosed with CAD and 6409 individuals without the condition. The incidence of coronary heart disease in our study sample of young and middle-aged individuals with hypertension was approximately 11.06%. The average age (standard deviation, SD) of those diagnosed with coronary heart disease was 51.16 (7.62) years, with 54.7% being male and 45.3% being female. The average age (standard deviation) of patients who did not have coronary heart disease was 46.26 (10.39) years, with 48.8% being male and 51.2% being female. Univariate logistic regression analyses were performed for each variable, and only those variables that showed statistically significant differences (*p* < 0.05) between the two groups were chosen. Among the laboratory test data, the following characteristics were included: arm circumference, upper arm length, body mass index (BMI), standing height, waist circumference, weight, diastolic, HDL, total protein, uric acid, total cholesterol, mercury, cadmium, lead, hematocrit (%), hemoglobin, lymphocyte percent (%), monocyte percent (%), platelet count (%), red cell count, red cell distribution width (%), albumin, alkaline phosphatase, alt, total calcium (mg/dL), cholesterol (mg/dL), creatinine (mg/dL), glucose (mg/dL), gamma-glutamyl transferase, iron (µg/dL), potassium, lactate dehydrogenase (LDH), and osmolality.

The questionnaire data included the following characteristics: Poverty Income Ratio, Frequency of Nighttime Urination, High Cholesterol Level, Urinary Leakage During Physical Activities, Overweight Status, Arthritis, Family History of Asthma and Diabetes, Sleep Disorders, Tobacco Use, Depression, Type of Occupation, Hours Worked Per Week, Average Daily Alcohol Consumption, Oral Health Status, Engagement in Moderate Recreational Activities, Average Sleep Duration, Current Weight, Weight One Year Ago, Weight Ten Years Ago, Heaviest Weight, and Age at Heaviest Weight.

Demographic data include the following characteristics: Gender, Education Level, Marital Status, and age.

Following the three-step feature selection process, the remaining features were used as the final predictors for developing the CAD risk assessment model for young and middle-aged individuals with hypertension. For detailed information on the included features, please refer to **Supplementary Table 1**.

### 3.2 Selection of CAD Risk Factors

To identify characteristic and consistent attributes from the data, we carried out a three-step approach for selecting features.

#### 3.2.1 AIC-based Stepwise Backward Elimination

**Fig. 1.**
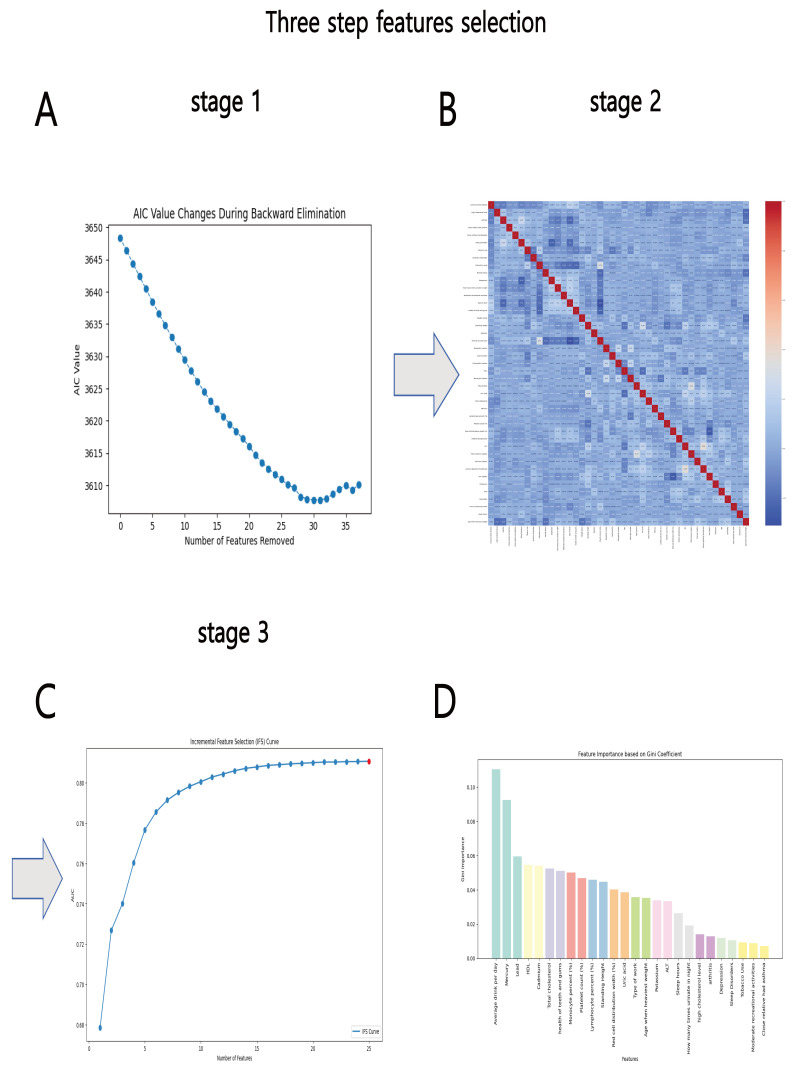
**Feature selection flow chart for coronary artery disease (CAD) risk assessment**. The three-step feature selection method is as follows: (A) Stage 1: Akaike Information Criterion (AIC) change curve from stepwise backward feature selection based on AIC; 59 features are retained. (B) Stage 2: Pearson correlation coefficient (PCC) heatmap of the remaining features. If the correlation between two features exceeds 0.4, the feature with the lower area under the curve (AUC) is removed, leaving 42 features. (C) Stage 3: Incremental feature selection (IFS) curve based on the AUC ranking of individual features. The red dot on the curve indicates the point of maximum AUC. (D) Bar chart showing the Gini index of the final 26 selected features. The height of each bar represents the importance (Gini coefficient) of the corresponding feature. ALT, alanine transaminase; HDL, high-density lipoprotein.

The initial analysis focused on determining the relationship between the incidence of CAD and the independent factors that had a *p*-value of less than 0.05 in the univariate logistic regression model. The process of feature selection was conducted by employing stepwise backward elimination, with the criterion for elimination being the minimal AIC. Fig. 1A displays the curve illustrating the variations in AIC, with a total of 59 features remaining after this stage.

#### 3.2.2 Pearson Correlation Coefficient Filtering

The second phase utilized the PCC to evaluate the correlation between the 59 features. The basis classifier used in this study was logistic regression (LR), and the classification performance of each feature was assessed using 5-fold cross-validation. The characteristics were prioritized according to their AUC values. If the correlation coefficient between two features was more than 0.4, the feature with the higher AUC was kept, while the feature with the lower AUC was discarded. Fig. 1B displays the heat map of the correlation coefficients for the 42 remaining attributes after this stage.

#### 3.2.3 Incremental Feature Selection

In the third step, 26 features were retained through IFS. The IFS curve is shown in Fig. 1C. This optimal subset includes: Average Drinks Per Day, Type of Work, Health of Teeth and Gums, High Cholesterol Level, Total Cholesterol, Arthritis, Tobacco Use, Mercury, Monocyte Percent (%), Lead, Sleep Disorders, Platelet Count (%), Frequency of Urination at Night, Standing Height, HDL, Red Cell Distribution Width (%), Potassium, Family History of Asthma, Engagement in Moderate Recreational Activities, ALT, Uric Acid, Sleep Hours, Lymphocyte Percent (%), Cadmium, Depression, and Age at Heaviest Weight. The Gini index for these 26 characteristics is provided in Fig. 1D.

### 3.3 Development and Validation of Five Machine Learning Models

#### 3.3.1 Performance of Baseline Models

**Fig. 2.**
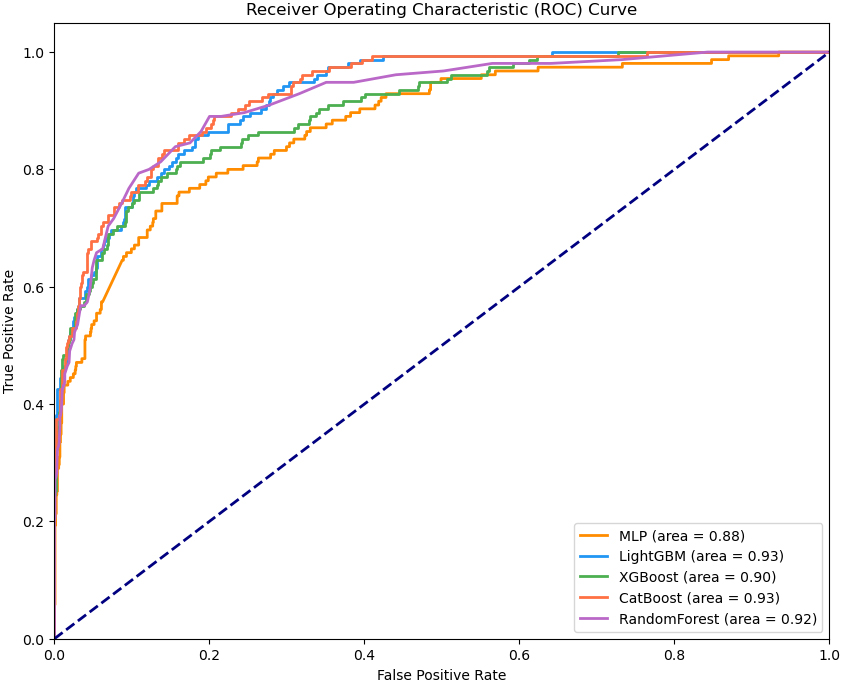
**The ROC curve of each model**. MLP, multilayer perceptron.

We utilized 26 optimal characteristics and applied five distinct algorithms—MLP, XGBoost, LightGBM, CatBoost, and random forest—to develop models for assessing CAD risk in young and middle-aged individuals with hypertension. We then compared the performance of these models on an internal validation set. The evaluation metrics, such as AUC, accuracy, precision, specificity, and F1 score, for the five models, are documented in Table 1, while the receiver operating characteristic (ROC) curves are displayed in Fig. 2. Of these five algorithms, the performance of the three methods is nearly identical, except for MLP and random forest. The LightGBM and CatBoost models outperformed the XGBoost model, having a slightly higher AUC of 0.93.

**Table 1.** **Performance metrics of machine learning algorithms for each model**.

Algorithm	AUC	Accuracy	Precision	Specificity	Recall	F1-Score
Random forest	0.91	0.93	0.93	0.99	0.35	0.51
XGBoost	0.91	0.93	0.76	0.97	0.52	0.62
LightGBM	0.92	0.93	0.80	0.99	0.59	0.68
CatBoost	0.94	0.92	0.78	0.98	0.49	0.60
MLP	0.87	0.92	0.75	0.99	0.42	0.54
Random forest (HPO)	0.92	0.93	0.92	0.99	0.40	0.56
XGBoost (HPO)	0.93	0.93	0.80	0.97	0.58	0.67
LightGBM (HPO)	0.91	0.93	0.88	0.99	0.64	0.74
CatBoost (HPO)	0.94	0.94	0.82	0.98	0.54	0.65
MLP (HPO)	0.89	0.93	0.78	0.99	0.50	0.61

HPO, hyperparameter optimization; MLP, multilayer perceptron; AUC, area under the curve.

Considering that the model is employed for early detection of CAD in young and middle-aged individuals with hypertension in healthcare settings, our focus was on improving sensitivity (recall) to ensure that patients with CAD are correctly identified. Enhancing the F1 score was also deemed advantageous. Among the five models, the LightGBM model attained the highest F1 score of 0.68 and a recall of 0.59.

#### 3.3.2 Hyperparameter Optimization and Final Model Selection

After conducting we observed that HPO using GA, XGBoost, LightGBM, and CatBoost showed very similar evaluation metrics. Among these models, LightGBM achieved the highest accuracy improvement, reaching 0.88; however, its F1 score did not show significant improvement. Therefore, we concluded that LightGBM demonstrated the best performance among these five models on the internal validation set. When the data is simple, and features are linearly independent, various algorithms tend to yield similar fitting results. However, in the current dataset, the LightGBM model exhibited slightly better performance, potentially due to its superior handling of large structured tabular data.

### 3.4 Predictive Variable Analysis

#### 3.4.1 Global Interpretation With SHAP Values

**Fig. 3.**
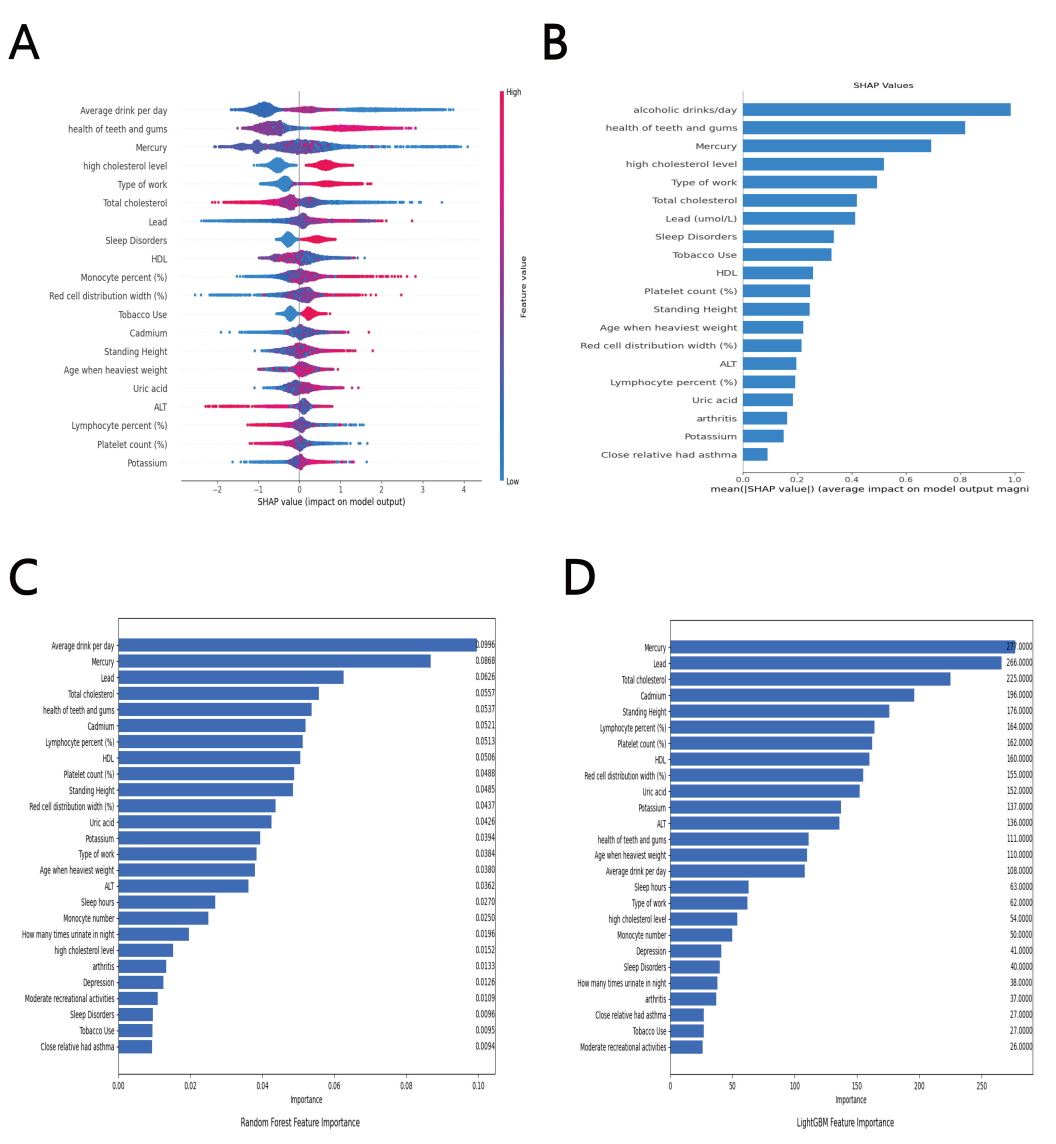
**Statistical results of risk factors for CAD**. (A) The mean absolute SHapley Additive exPlanations (SHAP) values. (B) Summary plot of the top 20 in prediction tasks by SHAP. (C) Features importance by random forest. (D) Features importance by LightGBM. ALT, alanine transaminase; HDL, high-density lipoprotein; CAD, coronary artery disease.

To comprehend the impact of features on the model, we performed an interpretability study utilizing SHAP. Fig. 3A presents a SHAP plot that illustrates all sampling points. The colors in the figure correspond to the magnitude of feature values, with red representing big values, blue representing low values, and purple representing values close to the mean. The exact SHAP absolute values are displayed in Fig. 3B. The magnitude of the values on the x-axis directly corresponds to their influence on the model. As illustrated in the diagram, the model is most significantly influenced by the average daily alcohol consumption.

Factors such as dental and gingival health, elevated cholesterol levels, job absenteeism or unemployment, exposure to lead, sleep disorders, monocyte count, red blood cell distribution width, smoking, cadmium exposure, height, uric acid levels, and potassium levels have been found to positively impact CAD. Conversely, CAD was total cholesterol, the proportion of lymphocytes, and platelet count negatively affect CAD. Interestingly, when mercury and HDL were at or above the average, their impact on CAD risk was modest. However, as their levels dropped below the average, their impact on CAD became significantly more pronounced.

#### 3.4.2 Feature Importance in Random Forest and LightGBM

The significance of the 26 features included in the RF and LightGBM models was evaluated (refer to Fig. 3C for RF and Fig. 3D for LightGBM). The rankings of feature importance obtained from both the LightGBM and RF algorithms showed a high degree of similarity, with the top 15 features being nearly identical and having similar importance rankings. According to the RF model, the three most influential factors affecting CAD are average daily alcohol consumption, mercury, and lead. In contrast, the LightGBM model identified mercury, lead, and total cholesterol as the three most influential features affecting CAD.

The Gini index ranked the following characteristics as the most significant: mercury, lead, total cholesterol, cadmium, standing height, percentage of lymphocytes, platelet count, HDL, red blood cell distribution width, uric acid, average daily alcohol consumption, and dental health.

#### 3.4.3 Individual Prediction Example

The analysis of individual predictions involves integrating personalized input data to examine how a specific prediction is generated for an individual. **Supplementary Fig. 2** illustrates a hypertensive young adult with CAD. The prediction model indicates a 92% probability that the individual is classified as having “CAD” (see **Supplementary Fig. 2A**), and an 8% probability of being classified as “non-CAD” (see **Supplementary Fig. 2B**). The waterfall plot displays the actual measurements of the features, except for average daily alcohol consumption, which contributed to the classification “CAD”. Additionally, explanatory power plots for individuals in the internal validation cohort are available in **Supplementary Fig. 2C**. The x-axis denotes each sample, while the y-axis reflects the contribution of each feature. An increase in the intensity of the red hue in each sample indicates a greater probability of identifying the sample as ‘CAD’.

### 3.5 Convenient Application for Clinical Utility

The final prediction model was implemented into a web application to facilitate its use across various healthcare scenarios (see **Supplementary Fig. 3**). This application automatically predicts the risk of CAD in individual young patients with hypertension, based on the input of relevant feature values. The Web application is available at https://prediction-of-coronary-heart-disease-htn-young-adults.streamlit.app/onlineaccess.

## 4. Discussion

This study aimed to develop a risk assessment model for CAD, specifically targeting young and middle-aged adults with hypertension. Among the models tested, LightGBM demonstrated the best performance, achieving an AUC of 0.93. These results suggest that our model can reliably identify individuals at elevated risk of CAD using only routine laboratory tests and simple questionnaire data. This approach is particularly suitable for early screening in primary healthcare settings or other healthcare facilities for young and middle-aged adults, compared to traditional CAD evaluation models. Additionally, it offers greater generalizability. It can also be used for early detection and monitoring of individual susceptibility, aiding in the community management of hypertension-related risk among young and middle-aged adults at high risk of coronary heart disease.

**Fig. 4.**
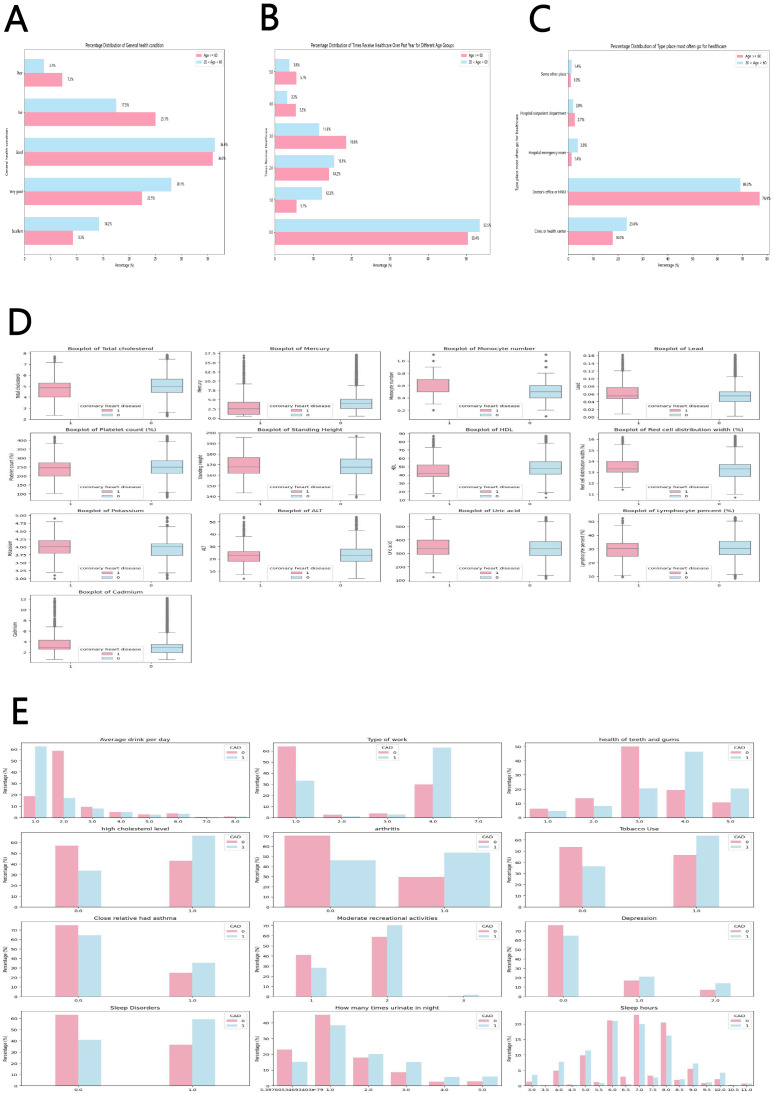
**Statistical results of hospital utilization and CAD risk factors in middle-aged and young adults**. (A) Percentage bar graphs of young and middle-aged people (18–60 years) and older adults (>60 years) who perceive their general health. (B) Percentage bar graphs of the number of times young and middle-aged people and older adults have received health care in the past year. (C) Percentage bar graphs of the types of healthcare settings most frequently visited by young and middle-aged people and older adults. (D) Statistical analysis of the 13 best continuous characteristics of patients with coronary artery disease and healthy individuals. (E) Statistical analysis of the 13 best discrete characteristics of patients with coronary artery disease and Statistical analysis of the 12 best discrete characteristics of healthy individuals. ALT, alanine transaminase; HDL, high-density lipoprotein; CAD, coronary artery disease.

Young and middle-aged adults often encounter challenges in achieving early prevention of CAD due to their utilization, restricted access to care, and a perception of being in excellent health. Fig. 4A displays a bar chart showing the percentage of young and middle-aged individuals who perceive their general health status compared to those over 60 years old. The chart reveals that the middle-aged group is more inclined to consider themselves in “Excellent” or “very good” health than the older population. Healthcare access can be quantified by the frequency of healthcare visits within the previous year. Fig. 4B demonstrates that 66% of individuals in the young and middle-aged group had either no visits or only one visit to a healthcare practitioner in the previous year. In contrast, a greater percentage of senior adults had three or more visits compared to the younger population. Hence, it is imperative to enhance the availability of options for this demographic to raise awareness of their CAD risk to avoid the dire consequences of untimely mortality. Moreover, Fig. 4C shows that a higher percentage of young and middle-aged adults attended clinics, health facilities, or hospital emergency departments more frequently than the elderly. This suggests that individuals in this age group may experience more severe repercussions when they get a disease since they have lower rates of hospital utilization and delayed diagnoses.

Routine blood and urine tests, combined with basic questionnaires, provide convenient and comprehensive data, making them well-suited for disease screening purposes. A series of traits were examined to forecast the likelihood of CAD. Fig. 4D,E illustrates the distribution of the most significant features, emphasizing the differences between patients with CAD and healthy individuals in the hypertensive young and middle-aged population. Multiple studies have shown a robust correlation between these characteristics and CAD. Numerous studies have demonstrated that elevated cholesterol is a significant risk factor for CAD [25], increasing mortality among hypertensive patients [26], while HDL serves as a protective factor. In the present study, total cholesterol and HDL levels were found to be low in the population with CAD. Fig. 4E shows that patients with CAD are more likely to have elevated cholesterol levels. The observed low total cholesterol levels in this population may be attributed to the common clinical co-occurrence of hypertension and dyslipidemia [27], which is often managed with lipid-lowering medications. Additionally, patients with CAD exhibit a high red blood cell distribution width (RDW), which is a novel independent marker for CVD, including heart failure, CAD, and myocardial ischemia [28]. Additionally, elevated RDW is associated with an increased myocardial scar load in patients with CAD.

The significant influence of average daily alcohol consumption on CAD risk is noteworthy. The model suggested that low levels of alcohol use were associated with reduced risk, while both heavy consumption and complete abstinence were linked to increased risk. This pattern aligns with prior research indicating a U-shaped relationship between alcohol consumption and cardiovascular outcomes. Specifically, excessive alcohol intake has been associated with elevated CVD risk, while moderate consumption may offer cardioprotective effects, particularly against CAD and ischemia-reperfusion injury [29]. A large cohort study also found a U-shaped relationship between the amount of alcohol consumed per week and CAD. Non-drinkers and individuals consuming more than 248 grams of alcohol per week had an approximately twofold increased risk of death compared to those consuming moderate amounts (10–80 grams of alcohol per week) [30]. The impact of ALT levels also demonstrated a non-linear trend. It is important to mention that ALT has a negligible impact on the risk of CAD when its levels are at or below the average, but it can have both beneficial and detrimental impacts on CAD risk when its levels are above the average. Prior research has established a correlation between raised ALT levels and a heightened likelihood of CAD and cardiovascular disease [31]. In the present study, the observed association between increased ALT levels and a decreased risk of CAD may be attributable to the correlation between elevated ALT and other CAD risk factors, such as high blood pressure, elevated total cholesterol, and triglyceride levels [32]. Given that the study cohort consisted of individuals with hypertension, the presence of elevated ALT levels may have influenced the assessment of ALT’s impact on the risk of CAD, thus introducing bias into the study’s conclusion.

**Fig. 5.**
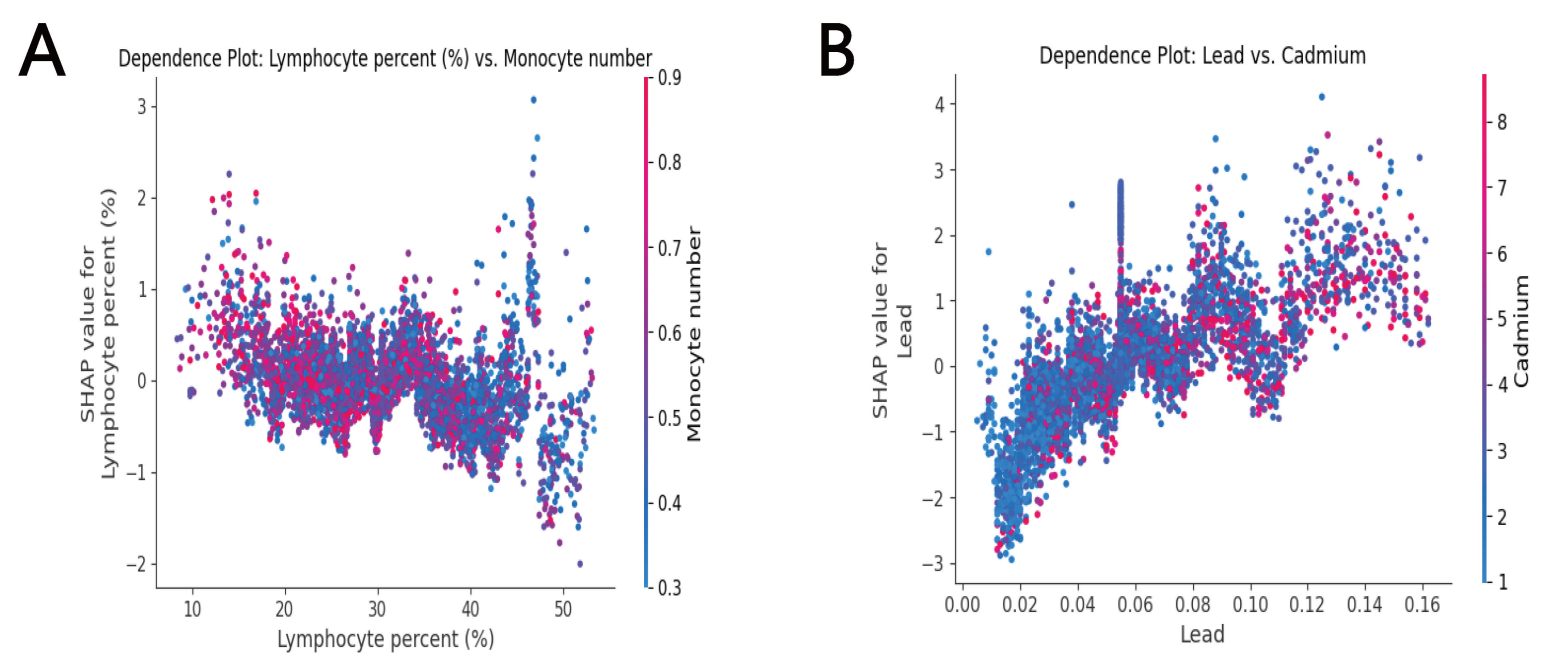
**SHapley Additive exPlanations (SHAP) interaction scatter plots for lead–cadmium and lymphocyte–monocyte feature pairs**. (A) A SHAP scatter plot for lead and cadmium, where the x-axis represents the actual value of lead, the y-axis represents the SHAP value of lead, and the color of the dots represents the magnitude of the cadmium value, can be used to show the interaction between features. (B) SHAP scatter plot of lymphocyte percentage and monocyte number.

Inflammation plays a crucial role in all stages of atherosclerosis. High platelet-lymphocyte ratio (PLR) levels are independently associated with the severity of coronary atherosclerosis [33]. Fig. 5A displays a SHAP scatter plot illustrating the numbers of lymphocytes and monocytes. A drop in the number of lymphocytes is accompanied by an increase in the number of monocytes, which further enhances the predictive value for CAD. Both lymphocytes and monocytes are implicated in inflammation, providing more evidence of a connection between CAD and inflammatory mechanisms. Interestingly, we observed that a high proportion of patients with CAD also had arthritis. Evidence indicates that patients with arthritis have a significantly increased risk of CAD [34], suggesting that CAD and arthritis may share similar pathological mechanisms and risk factors. Increased platelet activation has also been observed in individuals prone to depression or hostility, as well as those exposed to high levels of work-related stress [35]. Research has found that patients with CAD tend to have elevated levels of depression. This indicates that platelet activation may function as a connection between psychological stress and an increased chance of developing coronary issues. These data indicate that the use of anti-inflammatory medicine may have a potential impact on decreasing the likelihood of developing CAD.

Epidemiological evidence indicates a positive correlation between uric acid levels and coronary heart disease, as well as a link with poor prognosis for cardiovascular events [36]. The association of high uric acid levels and nocturnal urination with CAD suggests a link between CAD and kidney disease. Our model underscores the significance of various diseases associated with CAD, emphasizing their role in risk assessment and management.

Global public health has increasingly been impacted by exposure to environmentally hazardous metals of hydrogeological origin, including arsenic, lead, cadmium, mercury, and copper. This study identified a strong correlation between ambient hazardous metals and the occurrence of CAD. Individuals with CAD exhibited elevated concentrations of lead and cadmium, while levels of mercury were found to be minimal. Fig. 5B demonstrates that higher concentrations of lead along with increased levels of cadmium, improve the accuracy of predicting CAD. A meta-analysis has established a clear association between exposure to arsenic, lead, cadmium, and copper with a heightened risk of CVD and CAD [37]. Overall, the effects of mercury on cardiovascular health remain controversial. The observed low levels of mercury in patients with CAD may be attributable to the association of mercury exposure with fish consumption, which is known to have other cardiovascular benefits [38]. Consequently, the potentially harmful effects of mercury exposure might be counterbalanced. The results also emphasize the significance of environmentally toxic metals in contributing to the risk of CAD, beyond the impact of traditional behavioral risk factors.

Nevertheless, it is crucial to acknowledge that the scarcity of positive samples, caused by the significant imbalance in our dataset, leads to diminished recall and F1 scores. However, our methodology provides improved accessibility in healthcare facilities as compared to earlier CAD risk assessment methods. The model’s recall and overall F1 performance highlight its potential for early detection of high-risk individuals in primary care, where identifying true positives is crucial. Additionally, our model incorporates factors such as dental health, depression, and arthritis that were not considered in earlier models. This introduces a new aspect of population health management with CAD. Despite its promising performance, this study has several limitations. First, the model was trained and evaluated using cross-sectional data from the NHANES database, which limits the ability to infer causal relationships or assess long-term predictive performance. Second, currently lacks external validation using independent datasets from different populations or healthcare systems. Future studies should focus on prospective validation and longitudinal follow-up to evaluate real-world clinical utility and generalizability. Moreover, the abundance of missing values in our dataset may adversely affect the prediction accuracy; however, employing larger and higher-quality datasets in the future could help address this issue.

## 5. Conclusions

We developed a prediction model utilizing routine blood, urine, and basic questionnaire data to assess the risk of CAD in young and middle-aged individuals with hypertension. This model aims to facilitate early screening and reduce the risk of sudden cardiac death (SCD) in this high-risk population, which often experiences sudden onset and poor prognosis while utilizing healthcare resources sparingly. The study indicates that the LightGBM model exhibits the best predictive performance among the five machine learning models evaluated. Our model also highlighted several related diseases and the significance of environmental toxic metals in CAD, which may assist healthcare professionals in providing early diagnoses and personalized health management programs for individuals at risk of CAD. The final prediction model has been integrated into a web application to facilitate its use across various healthcare settings.

## Availability of Data and Materials

The data was obtained from NHANES (https://wwwn.cdc.gov/nchs/nhanes/Default.aspx).

## Supplementary Material

Supplementary material associated with this article can be found, in the online version, at https://doi.org/10.31083/RCM39006.
