# Supplementary material for: Development and Validation of an Explainable Prediction Model to Assess the Risk of Coronary Artery Disease in Young and Middle-Aged Individuals
Source: Rev Cardiovasc Med. 2025 Sep 23;26(9):39006. doi: 10.31083/RCM39006 (PMC12516760; doi:10.31083/RCM39006)
Supplement: Supplementary file 1 [file 2153-8174-26-9-39006-s1.docx]

# Supplementary Fig. 1. Flow chart of modeling.


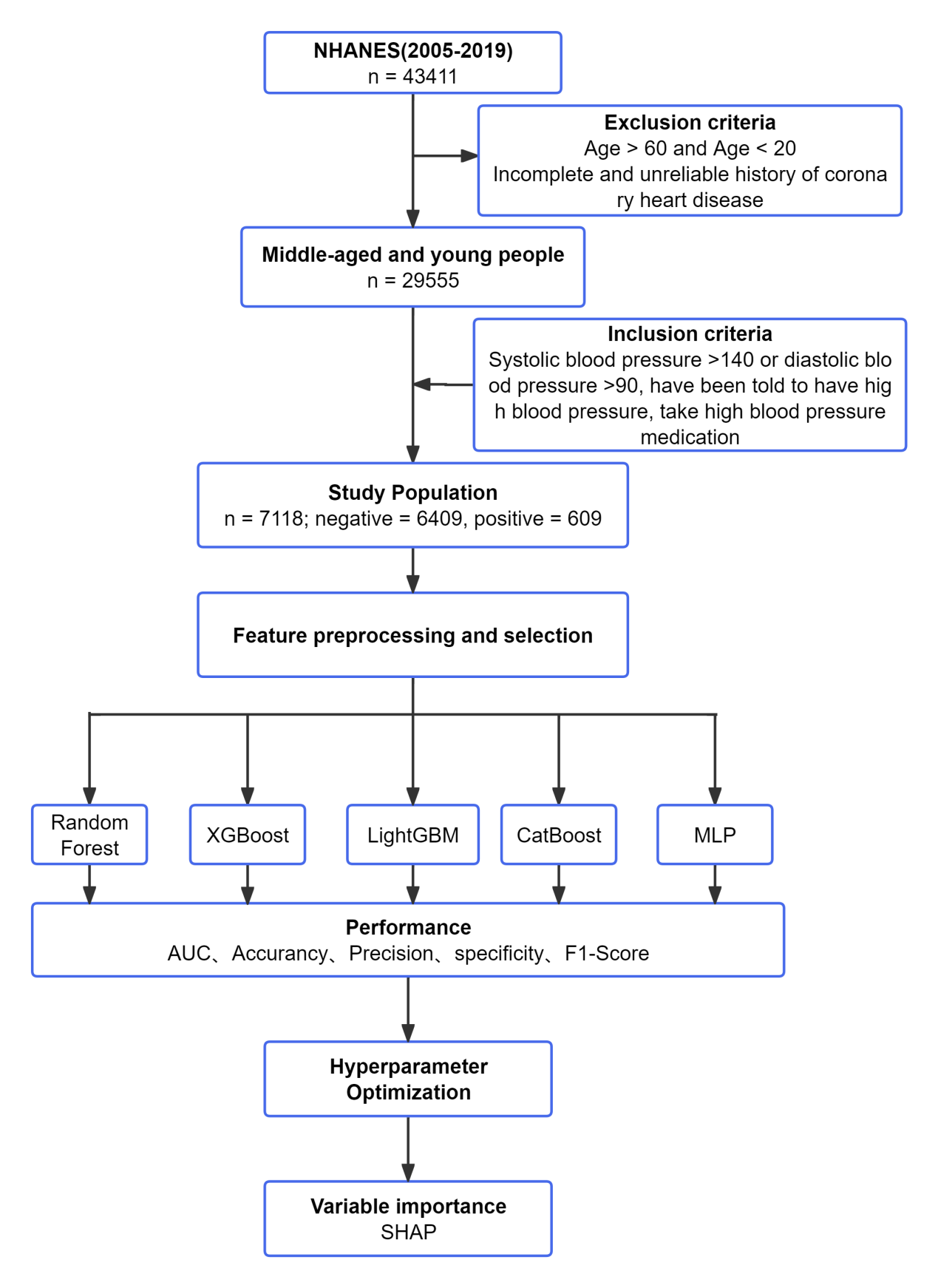


NHANES, the National Health and Nutrition Examination Survey; XGBoost, eetreme gradient boosting; LightGBM, light gradient-boosting machine; CatBoost,categorical boosting; MLP,Multilayer Perceptron; SHAP, Shapley additive explanations.

**Supplementary Table 1.** General characteristics of all model variables.

| **Variable** | **No coronary heart**  **disease(n=6409)** | **coronary heart disease(n=709)** | ***p*-Value** |
| --- | --- | --- | --- |
| Average drink per day |  |  | <0.001 |
| 1 | 1203(18.8%) | 443(62.5%) |  |
| 2 | 3763(58.7%) | 123(17.3%) |  |
| 3 | 602(9.4%) | 56(7.9%) |  |
| 4 | 324(5.1%) | 36(5.1%) |  |
| 5 | 184(2.9%) | 18(2.5%) |  |
| 6 | 242(3.8%) | 23(3.2%) |  |
| 7 | 36(0.6%) | 3(0.4%) |  |
| 8 | 55(0.9%) | 7(1.0%) |  |
| Type of work |  |  | <0.001 |
| Working at a job or business | 4101.0 (64.0%) | 234.0(33.0%) |  |
| Not working at a job or business | 1908.0(29.8%) | 446.0(62.9%) |  |
| health of teeth and gums |  |  | <0.001 |
| Very good | 876(13.7%) | 57(8.0%) |  |
| Good | 3206 (50.0%) | 146(20.6%) |  |
| Fair | 1244(19.4%) | 328(46.3%) |  |
| Poor | 684(10.7%) | 145(20.5%) |  |
| high cholesterol level |  |  | <0.001 |
| Yes | 3650(57.0%) | 240(33.9%) |  |
| No | 2759(43.0%) | 469(66.1%) |  |
| arthritis |  |  | <0.001 |
| Yes | 4509(70.4%) | 328(46.3%) |  |
| No | 1900 (29.6%) | 381(53.7%) |  |
| Tobacco Use |  |  | <0.001 |
| Yes | 3434(53.6%) | 258(36.4%) |  |
| No | 2975(46.4%) | 451(63.6%) |  |
| Sleep Disorders |  |  | <0.001 |
| Yes | 4065(63.4%) | 289(40.8%) |  |
| No | 2344(36.6%) | 420(59.2%) |  |
| How many times urinate in night |  |  | <0.001 |
| 1 | 1469(22.9%) | 108(15.2%) |  |
| 2 | 2895(45.2%) | 272(38.4%) |  |
| 3 | 548(8.6%) | 107(15.1%) |  |
| 4 | 169(2.6%) | 39(5.5%) |  |
| 5 | 182(2.8%) | 41(5.8%) |  |
| Close relative had asthma |  |  | <0.001 |
| Yes | 4809(75.0%) | 458(64.6%) |  |
| No | 1600(25.0%) | 251(35.4%) |  |
| Depression |  |  | <0.001 |
| 0 | 4874(76.0%) | 460(64.9%) |  |
| 1 | 1088(17.0%) | 150(21.2%) |  |
| 2 | 447(7.0%) | 99(14.0%) |  |
| Moderate recreational activities |  |  | <0.01 |
| Yes | 2632(41.1%) | 199(28.1%) |  |
| No | 3765(58.7%) | 499(70.4%) |  |
| Total cholesterol | 5.05±0.95 | 4.73±0.99 | <0.001 |
| Mercury | 4.60±3.18 | 3.61±3.31 | <0.001 |
| Lead | 0.06±0.03 | 0.06±0.03 | <0.001 |
| Platelet count | 253.32±57.47 | 241.33±59.80 | <0.001 |
| Cadmium | 3.33± 2.23 | 3.78 ± 2.44 | <0.001 |
| Standing Height | 168.07±9.97 | 168.96±10.07 | <0.001 |
| HDL | 49.16±12.46 | 45.65±12.47 | <0.001 |
| Red cell distribution width | 13.30±0.94 | 13.54±0.93 | <0.001 |
| Monocyte number | 0.55 ± 0.17 | 0.57 ± 0.17 | <0.05 |
| Sleep hours | 6.83±1.48 | 6.70±1.73 | <0.001 |
| Potassium | 3.94±0.32 | 3.99±0.33 | <0.001 |
| ALT | 23.83±9.02 | 23.08±8.43 | <0.001 |
| Uric acid | 336.73±79.36 | 345.22±80.89 | <0.001 |
| Lymphocyte percent | 30.82±7.80 | 29.72±7.95 | <0.001 |
| Age when heaviest weight | 40.51±11.32 | 42.78±10.98 | <0.001 |

For categorical variables,mean and percentages are given,Chi-square tests were used to assess inter-group differences in features.For continuous variables, mean and standard deviation are given,t tests were used to assess the inter-group differences in features.

**Supplementary Fig. 2.** Local model explanation by the SHAP method.

**
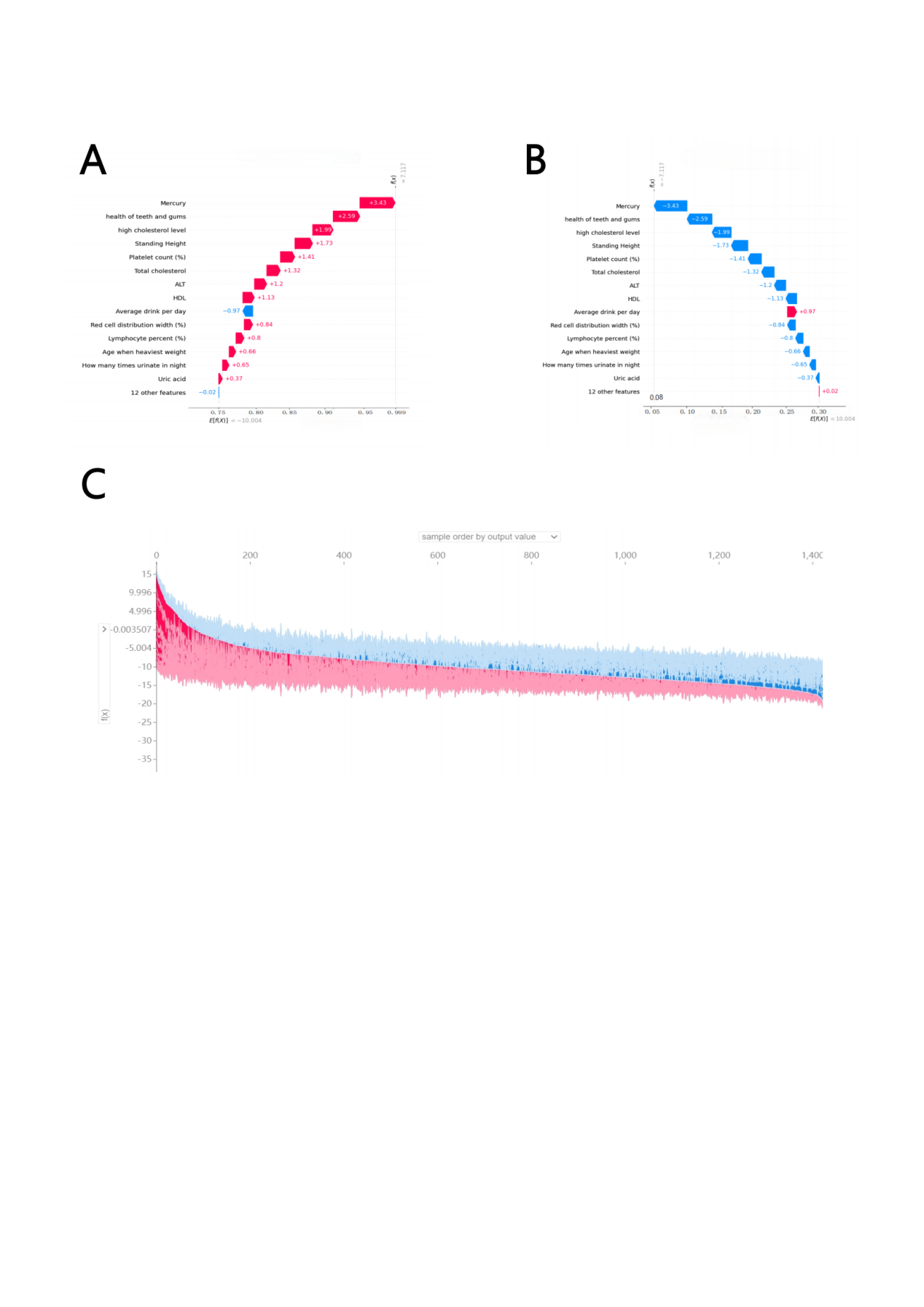
**

A Individual waterfall plot representing coronary heart disease. B Individual waterfall plot representing non-coronary heart disease.C Force plot for the internal validation set. Each patient was represented by the x-axis, while the features’ contributions were represented by the y-axis.

**Supplementary Fig. 3.** Convenient application for screening.


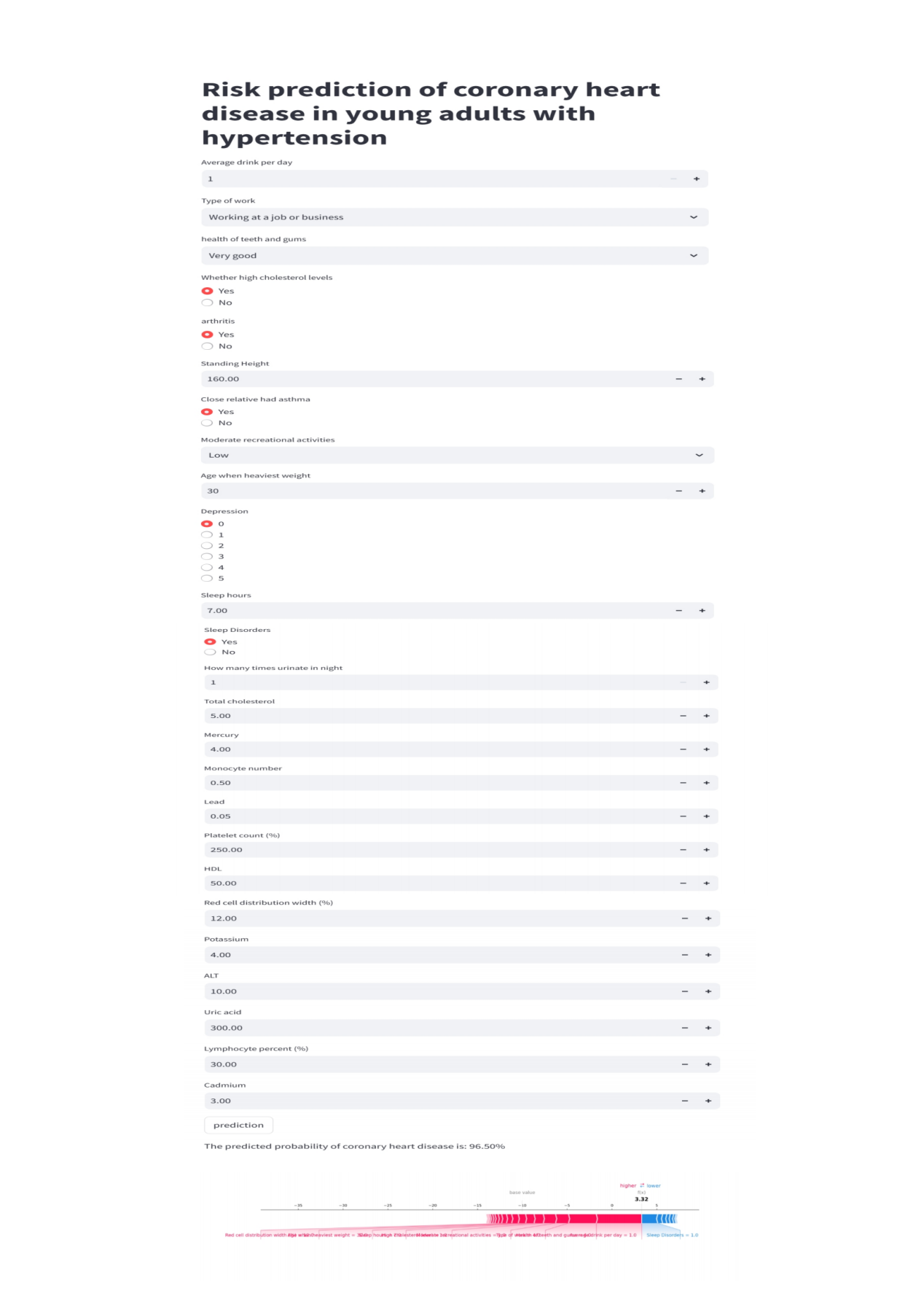


Enter the actual values of 26 features and the app automatically displays the probability of developing CAD. At the same time, the individual's force plot is shown at the bottom: the blue feature on the right is the feature that pushes the prediction into the "non-CAD" category, while the red feature on the left pushes the prediction into the "CAD" category.
